# Diagnostic approach to glomerular diseases: integrating clinical, laboratory, and histopathological assessments

**DOI:** 10.1097/MS9.0000000000003637

**Published:** 2025-07-22

**Authors:** Chukwuka Elendu, Chiemezie E. Ejiogu, Elijah O.A. Adetunji, Linda S. Mensah, Treasure A. Chinuokwu, Tochukwu W. Okahia, Abolore Aminat Ajakaye, Boluwatife D. Oshin, Varun Tyagi, Lordsfavour I. Anukam, Chiamaka O. Oguoma, Aminu A. Dogondaji, Chukwuemeka C. Njoku

**Affiliations:** a Federal University Teaching Hospital, Owerri, Nigeria; b Afe Babalola University, Ado-Ekiti, Nigeria; c Kharkiv National Medical University, Kharkiv, Ukraine; d V.N. Karazin Kharkiv National University, Kharkiv, Ukraine; e Doncaster and Bassetlaw Teaching Hospitals NHS Foundation Trust, Doncaster, United Kingdom; f University Hospital Coventry and Warwickshire, Coventry, United Kingdom; g Bogomolets National Medical University, Kyiv, Ukraine; h Babcock University, Ilishan-Remo, Nigeria; i Basildon Hospital, Basildon, United Kingdom; j International University of the Health Sciences, Basseterre, Saint Kitts and Nevis; k Abia State University Teaching Hospital, Aba, Nigeria; l Usmanu Danfodiyo University, Sokoto, Nigeria; m Nnamdi Azikiwe University Teaching Hospital, Nnewi, Nigeria

**Keywords:** diagnostic approach, glomerular diseases, kidney biopsy, nephrology practice, resource-limited settings

## Abstract

**Background:**

Glomerular diseases are a significant contributor to chronic kidney disease globally, accounting for up to 30% of end-stage kidney disease cases. Diagnostic accuracy is crucial for appropriate management but remains challenging, especially in low-resource settings.

**Objective:**

Our paper addresses the diagnostic approach to glomerular diseases by integrating clinical evaluation, laboratory findings, imaging techniques, and histopathological assessments. It also highlights regional disparities and systemic barriers affecting diagnostic capacity.

**Methods:**

We conducted a narrative review of published literature, synthesizing data from peer-reviewed articles, international registries, and survey reports to explore current diagnostic approaches to glomerular diseases. We searched databases, including PubMed, Scopus, and Google Scholar, for relevant studies published between January 2020 and December 2024.

**Results:**

Biopsy access was markedly limited in Africa, with only 49.7% of respondents able to perform biopsies in more than 10% of indicated cases, compared to 95.7% in Asia. Immunofluorescence and electron microscopy were unavailable or underutilized in many centers, especially in Africa. Serological testing for autoimmune glomerulopathies and the availability of key immunosuppressive therapies were also significantly constrained. These limitations frequently led to empiric treatment without a definitive diagnosis, raising concerns about mismanagement and healthcare inequities.

**Conclusion:**

A multidisciplinary diagnostic approach remains essential for managing glomerular diseases. However, significant disparities in access to biopsy, nephropathology, and serological testing hamper optimal care in many regions. Investment in diagnostic infrastructure, clinician training, and health policy reforms are critical for improving global kidney health outcomes.

## Introduction and background

Chronic kidney disease is a global health burden characterized by progressive loss of renal function over time, affecting millions of individuals worldwide^[1]^, with glomerular diseases constituting a significant subset of these cases. It is estimated that chronic kidney disease affects approximately 10–15% of the global adult population, with glomerular disorders contributing to up to 25–30% of end-stage kidney disease cases in various regions^[2,3]^. The burden of glomerular disease is particularly notable in low- and middle-income countries, where limited access to healthcare and delayed diagnosis contribute to poor outcomes^[4]^. Studies indicate geographic and ethnic variability in glomerular disease patterns; for example, IgA nephropathy is more prevalent in East Asia, while focal segmental glomerulosclerosis is more common among individuals of African descent^[3-5]^.

**HIGHLIGHTS**

- Limited access to biopsy and pathology services impedes accurate diagnoses.
- Financial constraints lead to empiric treatment over evidence-based therapy.
- Major diagnostic and drug access gaps persist in low-resource settings.

The rising prevalence of glomerular diseases is attributed to aging populations, increasing rates of diabetes and hypertension, as well as improvements in diagnostic capacity^[1,3]^. Secondary glomerular diseases, such as lupus nephritis and diabetic nephropathy, also demonstrate region-specific trends linked to underlying systemic conditions. These epidemiologic insights highlight the importance of context-specific strategies for diagnosis, monitoring, and management^[1,4]^. Despite advancements in nephrology, the diagnostic journey for patients with glomerular disease often remains fragmented – especially when clinical signs, lab results, and biopsy findings don’t immediately align^[6-9]^. Our paper was developed to address that challenge by offering a structured diagnostic roadmap emphasizing integration: how the clinical picture, laboratory data, imaging, and histopathology can – and should – work together to sharpen diagnostic clarity.

Our target audience includes pediatric and adult healthcare providers – internists, pediatricians, nephrologists, and general physicians – who are often the first to encounter these patients. Our paper serves as a diagnostic guide, synthesizing current evidence across age groups to support a more unified and evidence-informed clinical approach.

## Materials and methods

Our paper synthesized recent literature on diagnostic approaches to glomerular diseases by integrating findings from observational studies, clinical trials, case reports, and expert guidelines. We conducted a literature search using Google Scholar and Refseek, covering studies published between January 2020 and December 2024. The search terms included “glomerular diseases,” “diagnostic approach,” “renal biopsy,” “glomerulonephritis diagnosis,” and “urine analysis,” with Boolean operators employed to optimize search precision.

Studies were included if they discussed diagnostic methods specific to glomerular diseases, detailed relevant methodologies such as biopsy, imaging, and serologic testing, or offered authoritative diagnostic guidelines. Articles were excluded if they focused exclusively on treatment, lacked methodological depth, or were not published in English.

Data from eligible studies were extracted into a structured format, capturing study design, diagnostic techniques evaluated, populations studied, outcomes, and conclusions. Any inconsistencies were resolved through consensus among the authors. The methodological quality of the included studies was appraised using adapted PRISMA criteria, emphasizing clarity of diagnostic strategies and clinical applicability. Studies with substantial methodological flaws or a high risk of bias were excluded.

Findings were synthesized qualitatively, highlighting different diagnostic modalities’ comparative effectiveness, strengths, and limitations. Additionally, consensus guidelines from major nephrology organizations were incorporated to contextualize current standards and innovations in diagnosing glomerular diseases.

## Results

Two hundred ninety-eight kidney care providers from 33 countries responded to the survey. Respondents included 159 (53.3%) from Asia and 133 (44.6%) from Africa. Twenty-one participants (7%) were from low-income countries, 260 (87.3%) were from lower-middle-income countries, and 17 (5.7%) were from upper-middle-income countries^[10]^. Table 1 summarizes the demographic and professional characteristics of the respondents. The majority (169; 63%) were involved in adult nephrology care, while 92 (34%) provided care to both adult and pediatric patients; only 8 (3%) were exclusively pediatric nephrologists. A higher proportion of African respondents worked in academic institutions than their Asian counterparts (74.8% vs. 54.7%)^[11]^. Overall, 115 (38.5%) respondents reported receiving partial or complete training outside their current country of practice, with a higher prevalence in Africa (45.8%) than in Asia (30.8%). The primary motivations cited were the lack of local training opportunities and the desire to acquire specialized clinical or research skills.

**Table 1:** Respondent characteristics

	Total	Africa	Asia	Eastern Europe
Age distribution (yrs)	(*N* = 269)	(*N* = 104)	(*N* = 159)	(*N* = 6)
< 30	3 (1.1%)	0 (0.0%)	3 (1.9%)	0(0.0%)
30–39	93 (34.6%)	33 (31.8%)	58 (36.5%)	2(33.3%)
40–49	98 (36.5%)	49 (47.1%)	48(30.2%)	1(16.7%)
50–59	48 (17.8%)	16 (15.4%)	30 (18.8%)	2(33.3%)
>60	27 (10.0%)	06 (5.7%)	20 (12.6%)	1(16.7%)
Work experience in yrs	(*N* = 269)	(*N* = 104)	(*N* = 159)	(*N* = 6)
< 5 yrs	55 (20.4%)	20 (19.2%)	34 (21.4%)	1(16.7%)
5–10	89 (33.1%)	42 (40.4%)	45 (28.3%)	2 (33.3%)
11–20	68 (25.3%)	26 (25.0%)	42 (26.4%)	0 (0.0%)
More than 20	57 (21.2%)	16 (15.4%)	38 (23.9%)	3 (50%)
Type of practice	(*N* = 269)	(*N* = 104)	(*N* = 159)	(*N* = 6)
Academic/university hospital	180 (66.9%)	88 (84.6%)	87 (54.8%)	5 (83.3%)
Private hospital	58 (21.6%)	7 (6.7%)	50 (31.4%)	1 (16.7%)
Solo practice	19 (7.0%)	4 (3.9%)	15 (9.4%)	0 (0.0%)
Group practice	12 (4.5%)	05 (4.8%)	07 (4.4%)	0 (0.0%)
Who performs kidney biopsy	(*N* = 269)	(*N* = 104)	(*N* = 159)	(*N* = 6)
Nephrologist	230 (85.5%)	84 (80.8%)	145 (91.2%)	1 (16.6%)
Others	15 (5.6%)	03 (2.9%)	08 (5.0%)	4 (66.8%)
Biopsy not performed	24 (8.9%)	17 (16.3%)	06 (3.8%)	1 (16.6%)
	Total	Africa	Asia	Eastern Europe

Data represents frequencies and percentages of respondents’ demographic and clinical characteristics – source: Ramachandran et al, Kidney Int Rep. 2022;7(10):2141–2149. doi: 10.1016/j.ekir.2022.07.002.

### Kidney biopsy practices

Biopsy services were unavailable at the institutions of 35 (11.8%) respondents, while in 18 (6.1%) cases, biopsies were performed by non-nephrologists, such as radiologists. Among the respondents, 72 (28.8%) reported that less than 10% of patients who required a biopsy for suspected glomerular disease were able to receive one. In comparison, 98 (39.2%) stated that more than 50% of such patients underwent the procedure^[2,3]^ (Table 2). A significant regional disparity was observed: 50.3% of respondents in Africa could perform biopsies in less than 10% of patients, compared to only 4.3% in Asia. The leading barriers to biopsy were cost (45.7%), lack of nephropathology services (40%), and inadequate training (5.7%).

**Table 2:** Kidney biopsy details

	Total	Africa	Asia	Others
Proportion getting kidney biopsy when indicated	(*N* = 250)	(*N* = 131)	(*N* = 113)	(*N* = 6)
<10%	72 (28.8%)	66 (50.4%)	05 (4.5%)	1 (16.7%)
10–25%	48 (19.2%)	30 (22.9%)	17 (15.0%)	1 (16.7%)
26–50%	32 (12.8%)	14 (10.7%)	17 (15.0%)	2 (33.3%)
>50%	98 (39.2%)	21 (16.0%)	74 (65.5%)	2 (33.3%)
No of kidney biopsies in a month	(*N* = 259)	(*N* = 101)	(*N* = 153)	(*N* = 5)
Not done	0 (0%)	0 (0%)	0 (0%)	0 (0%)
0–5	117 (45.2%)	67 (66.3%)	49 (32.0%)	1(20%)
5–10	62 (23.9%)	21 (20.8%)	39 (25.5%)	2 (40%)
More than 10	80 (30.9%)	13 (12.9%)	65 (42.5%)	2 (40%)
Mean turn-around time (days)	(*N* = 257)	(*N* = 99)	(*N* = 153)	(*N* = 5)
< 3	48 (18.7%)	05 (5.0%)	42 (27.4%)	1(20%)
3–7	96 (37.3%)	17 (17.2%)	78 (51.0%)	1(20%)
8–14	72 (28.0%)	42 (42.4%)	28 (18.3%)	2 (40%)
>14	41 (16.0%)	35 (35.4%)	05 (3.3%)	1 (20%)
Location of the nephro-pathology service	(*N* = 253)	(*N* = 97)	(*N* = 151)	(*N* = 5)
In my hospital	106 (41.9%)	44 (45.4%)	62 (41.0%)	0 (0%)
In my city	54 (21.3%)	11 (11.3%)	38 (25.2%)	5 (100%)
Another city	54 (21.3%)	14 (14.4%)	40 (26.5%)	0 (0%)
Overseas	39 (15.5%)	28 (28.9%)	11 (7.3%)	0 (0%)
Biopsies processed	(*N* = 246)	(*N* = 89)	(*N* = 152)	(*N* = 5)
LM only	41 (16.7%)	38 (42.7%)	03 (2.0%)	0 (0%)
LM and IF	98 (39.8%)	25 (28.1%)	69 (45.4%)	4 (80%)
LM, IF, and EM	107 (43.5%)	26 (29.2%)	80 (52.6%)	1 (20%)
Proportion of biopsies evaluated by IF/IHC	(*N* = 252)	(*N* = 94)	(*N* = 153)	(*N* = 5)
<10%	59 (23.5%)	52 (55.4%)	06 (3.9%)	1 (20%)
11–50%	20 (7.9%)	10 (10.6%)	10 (6.5%)	0 (0%)
50–75%	16 (6.3 %)	07 (7.4%)	09 (5.9%)	0 (0%)
>75%	157 (62.3%)	25 (26.6%)	128 (83.7%)	4 (80%)
Proportion of biopsies evaluated by EM	(*N* = 243)	(*N* = 90)	(*N* = 148)	(*N* = 5)
<10%	167 (68.7%)	73 (81.2%)	90 (60.8%)	4 (80%)
11–50%	29 (11.9%)	04 (4.4%)	25 (16.9%)	0 (0%)
50–75%	16 (6.6%)	03 (3.3%)	13 (8.8%)	0 (0%)
>75%	31 (12.8%)	10 (11.1%)	20 (13.5%)	1 (20%)

LM, IF, EM, and IHC refer to light microscopy, immunofluorescence, electron microscopy, and immunohistochemistry, respectively—source: Ramachandran et al, Kidney Int Rep. 2022;7(10):2141–2149. doi: 10.1016/j.ekir.2022.07.002.

Biopsy volumes were lower in Africa than in Asia, as shown in Table 2. 160 (64%) respondents had access to a nephropathologist within the same hospital or city. In comparison, 39 (15.6%) had to send samples overseas for evaluation. The turnaround time for biopsy reports was under 7 days for 144 (57.6%) respondents; however, 41 (16.4%) experienced delays exceeding 14 days. Report delays were more prevalent in African centers (>7 days in 78% vs. 22% in Asia). While 205 (83.3%) respondents had access to immunofluorescence (IF), only 107 (43.5%) had access to electron microscopy (EM). Notably, 42.7% of African respondents reported access only to light microscopy, in contrast to 2% in Asia^[10]^.

Among those with IF capabilities, 59 (23.4%) reported that less than 10% of biopsies were evaluated using this technique, whereas 157 (62.3%) could use IF in over 75% of their cases. In Africa, 55% of centers performed IF in less than 10% of biopsies, compared to just 3.9% in Asia. EM usage was low across both continents, with 69% of respondents reporting use in less than 10% of biopsies^[10]^.

### Access to serological testing

Respondents rated the difficulty of accessing key serological assays on a scale from 0 (no difficulty) to 5 (unable to access). For antinuclear antibody testing, 171 (57.7%) experienced no difficulty, while 50 (16.8%) reported severe difficulty. For antineutrophil cytoplasmic antibodies, 40.2% had no difficulty, and 24.9% faced severe limitations. Anti-glomerular basement membrane antibody testing was not specifically described. Still, anti-M-type phospholipase A2 receptor (PLA2R) antibody testing was readily accessible to only 25.7% of respondents, while 36.4% faced severe challenges obtaining it. African centers were more likely to report difficulty accessing all serological tests than those in Asia^[9,10]^.

### Therapeutic access and treatment practices

Availability of essential medications varied widely. Fifty-seven (19.2%) respondents reported limited or no access to angiotensin-converting enzyme inhibitors and angiotensin receptor blockers (Table 3). Severe limitations or unavailability were reported for diuretics (21.3%), glucocorticoids (21.3%), azathioprine (22.3%), cyclophosphamide (24.7%), calcineurin inhibitors (25%), mycophenolate mofetil (23.6%), and rituximab (38.5%). These challenges were predominantly reported in African countries, although access to rituximab was similarly limited across both continents^[10,12]^.

**Table 3:** Pharmacological interventions in glomerular diseases

Drug class	Common drugs	Mechanism of action	Indications	Dosage	Side effects	Monitoring parameters	Clinical considerations
Corticosteroids	Prednisone, Methylpred.	Anti-inflammatory, immunosuppress.	Nephrotic syndrome, lupus nephritis	0.5–1 mg/kg/day	Hypertension, hyperglycemia, osteoporosis	Blood glucose, BP, bone density	Taper slowly to avoid adrenal insufficiency
Calcineurin Inhibitors	Cyclosporine, Tacrolimus	Inhibit T-cell activation	FSGS, membranous nephropathy	2–5 mg/kg/day	Nephrotoxicity, hyperkalemia	Serum creatinine, potassium, and trough levels	Monitor kidney function regularly
Antimetabolites	Mycophenolate mofetil	Inhibits purine synthesis	Lupus nephritis, IgA nephropathy	1–2 g/day	GI upset, leukopenia	CBC, renal function	Consider steroid-sparing regimens
RAAS Inhibitors	ACE inhibitors, ARBs	Reduce proteinuria, BP	Diabetic nephropathy, FSGS	Lisinopril: 10–40 mg/day	Hyperkalemia, cough	Serum potassium, renal function	Dual RAAS blockade not recommended
Alkylating Agents	Cyclophosphamide	Crosslinks DNA inhibits B cells	Severe lupus nephritis, vasculitis	1–3 mg/kg/day	Hemorrhagic cystitis, infertility	CBC, urinalysis	Use MESNA for bladder protection
Anti-platelet Agents	Aspirin, Clopidogrel	Inhibit platelet aggregation	Antiphospholipid syndrome, vasculitis	Aspirin: 81–325 mg/day	GI bleeding, thrombocytopenia	CBC, GI symptoms	Combine with PPI in high-risk patients
Biologics	Rituximab	B-cell depletion	Refractory lupus nephritis, ANCA vasculitis	375 mg/m^2^ weekly ×4	Infusion reactions, infections	CBC, CD19 + B-cell count	Pre-medicate with steroids/antihistamines
Diuretics	Furosemide, Spironolactone	Reduce fluid overload	Edema in nephrotic syndrome	Furosemide: 20–80 mg/day	Hypokalemia, dehydration	Electrolytes, renal function	Combine with potassium-sparing diuretics carefully

Dosages may vary based on patient condition and response. The side effects listed are not exhaustive and may differ among patients. Monitoring parameters is essential for managing adverse effects and ensuring efficacy. Clinical considerations include patient-specific factors and potential interactions with other medications – source: Authors’ Creations.

Barriers to optimal care were widespread. 54.3% of respondents frequently reported delayed patient presentation, while 30% noted that diagnostic workups were often unfeasible. High treatment costs (40.5%), medication unavailability (25%), and religious or cultural barriers (8.4%) were also cited. These challenges were more frequently encountered by respondents in Africa^[10]^.

The financial burden was significant, with 175 (59.3%) respondents reporting that over 75% of patients bore the full cost of diagnosis and treatment out-of-pocket. An additional 44 (14.9%) reported out-of-pocket payments for 50–74% of their patient population. These patterns were consistent across both continents^[13]^.

Limited diagnostic access led to empiric treatment strategies. Over one-third (104; 35.6%) of respondents reported treating more than 25% of their patients with high-dose oral corticosteroids (>1 mg/kg/day of prednisolone) without definitive diagnoses. Empiric use of immunosuppressants – including azathioprine, cyclophosphamide (oral and intravenous), calcineurin inhibitors, and mycophenolate mofetil – was prevalent across both African and Asian centers^[10,13]^.

## Discussion

**Figure 1.**
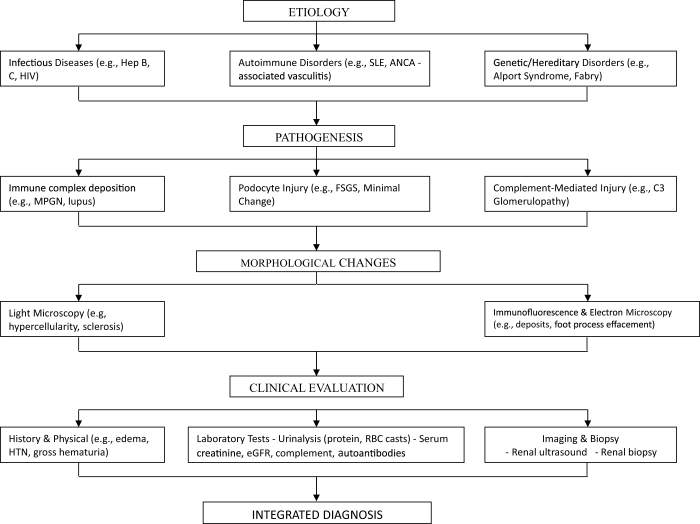
Overview of etiology, pathogenesis, and clinical evaluation of glomerular diseases.

Glomerular diseases constitute a significant burden in nephrology, accounting for a considerable proportion of chronic kidney disease and end-stage renal disease cases globally. Accurate diagnosis is essential for effective management and prognosis, given the diversity of etiologies and overlapping clinical presentations (See Fig. 1) ^[1-3]^. The complexity of glomerular diseases necessitates an integrated diagnostic approach that combines clinical assessment, laboratory testing, imaging studies, and histopathological evaluation. A literature synthesis across these domains reveals a compelling need for a multidimensional strategy that enhances diagnostic precision and informs therapeutic decisions.

This flowchart demonstrates how diverse etiologies converge on common pathogenic mechanisms, leading to characteristic morphologic changes, which are evaluated through a structured clinical approach – source: Authors’ Creations.

The epidemiology of glomerular diseases varies geographically, with differences attributed to genetic, environmental, socioeconomic, and healthcare system factors. In developed countries, conditions such as membranous nephropathy and focal segmental glomerulosclerosis (FSGS) are predominant, whereas post-infectious glomerulonephritis and lupus nephritis are more common in developing regions. Recent global health reports indicate a rising prevalence of glomerular diseases, paralleling increases in diabetes, hypertension, and autoimmune disorders^[1,4,5]^. This trend underscores the urgency for early and accurate diagnosis to mitigate long-term renal damage and systemic complications. Clinical evaluation remains the cornerstone of initial assessment. Patients typically present with nonspecific symptoms such as edema, hematuria, and hypertension, necessitating a high index of suspicion. The history should explore risk factors, including recent infections, autoimmune conditions, malignancy, and drug exposure^[6-8]^.

Physical examination may reveal signs suggestive of nephrotic or nephritic syndrome. The literature emphasizes the diagnostic value of structured clinical pathways that guide initial evaluation and determine the need for further testing. Notably, pediatric populations and elderly patients often present with atypical features, warranting tailored diagnostic algorithms. Laboratory investigations are essential for confirming clinical suspicion and narrowing differential diagnoses. Basic tests such as urinalysis, serum creatinine, and estimated glomerular filtration rate provide foundational information. Quantifying proteinuria using spot urine protein-to-creatinine ratio or 24-h urine collection helps differentiate nephrotic from non-nephrotic states. Advanced serological tests, including antinuclear antibody (ANA), anti-double stranded DNA, antineutrophil cytoplasmic antibodies (ANCA), anti-glomerular basement membrane (anti-GBM) antibodies, and anti-phospholipase A2 receptor (anti-PLA2R) are critical for identifying autoimmune and primary glomerulopathies^[9-11]^.

Literature suggests that a panel-based approach increases diagnostic yield and informs biopsy decisions. Moreover, emerging biomarkers such as soluble urokinase receptor (suPAR) and complement levels offer insights into pathophysiology and disease activity. Imaging studies complement laboratory tests by providing structural and functional information. Renal ultrasound is the most commonly employed modality, valued for its non-invasiveness, availability, and ability to detect kidney size, echogenicity, and structural abnormalities. Imaging can rule out obstructive uropathy or renal vein thrombosis in acute presentations. Doppler ultrasound helps assess renal blood flow and venous patency. Advanced imaging techniques like magnetic resonance imaging (MRI) and computed tomography (CT) are occasionally used in complex cases or when malignancy is suspected^[2,12,13]^. However, the literature advises cautious use due to cost, radiation exposure, and contrast nephropathy risks. Imaging plays a pivotal role in determining biopsy safety and guiding interventional procedures. Histopathological examination of kidney tissue remains the gold standard for definitive diagnosis. Percutaneous renal biopsy enables detailed analysis using light, immunofluorescence, and electron microscopy. These modalities allow the classification of glomerular diseases into categories such as minimal change disease, FSGS, membranous nephropathy, and various forms of immune complex-mediated glomerulonephritis^[14–16]^.

Biopsy findings confirm diagnosis, provide prognostic information, and guide treatment. Recent literature highlights interobserver variability and the importance of standardized reporting systems such as the Oxford Classification for IgA nephropathy and the ISN/RPS classification for lupus nephritis. Additionally, biopsy timing is crucial; delayed biopsy may lead to irreversible damage, while premature biopsy can pose unnecessary risk. Emerging diagnostics and digital pathology are transforming the landscape of glomerular disease diagnosis^[17,18]^. Multiplex immunoassays, proteomics, and genomics offer non-invasive and early detection opportunities. Studies have demonstrated the utility of urinary exosome analysis and gene expression profiling in distinguishing disease subtypes and predicting outcomes. Artificial intelligence (AI) and machine learning algorithms are being developed to interpret histological images, enhance diagnostic accuracy, and reduce pathologist workload. Digital pathology platforms facilitate remote consultations and training, which is particularly beneficial in underserved regions^[19–21]^. Although still early, these innovations promise to complement traditional diagnostics and personalize patient care.

Differential diagnosis of glomerular diseases is often challenging due to overlapping clinical and laboratory features. Conditions such as hypertensive nephrosclerosis, diabetic nephropathy, and interstitial nephritis can mimic glomerular pathology^[22]^. Moreover, secondary glomerular diseases associated with infections, malignancies, or systemic autoimmune disorders require comprehensive evaluation to identify underlying causes. Literature supports diagnostic algorithms incorporating clinical presentation, laboratory data, and response to empirical therapy. In complex cases, multidisciplinary team discussions involving nephrologists, pathologists, rheumatologists, and infectious disease specialists improve diagnostic accuracy and patient outcomes^[10,23,24]^.

### Barriers to biopsy and ancillary diagnostics

A central finding is the underutilization of kidney biopsy – considered the gold standard for diagnosing glomerular pathology – due to structural, financial, and educational barriers. Nearly one-third of respondents indicated that less than 50% of patients suspected of having glomerular disease underwent biopsy. Over 50% of participants also reported moderate-to-severe difficulty accessing serological tests vital for diagnosis. These limitations were significantly more common in African settings. While over three-quarters of responding nephrologists had formal training in kidney biopsies, the proportion of actively performing biopsies was lower in Africa. This discrepancy may stem from disparities in infrastructure, availability of biopsy needles, or support systems despite training. A trend analysis by Okani et al revealed a decline in biopsy procedures in Nigeria from 10–20 per year before 1993 to just 1–10 annually by 2011, even as access to kidney replacement therapy was improving during the same period. The decline was attributed primarily to out-of-pocket healthcare costs and the lack of skilled personnel^[8,9]^.

### Access to histopathology and the role of telemedicine

Access to nephropathology services remains inconsistent. In many LMICs, biopsy samples must be shipped to distant cities or even other countries, leading to delays that can be catastrophic in rapidly progressive conditions. As Table 2 shows, nearly 30% of respondents indicated the need to ship biopsies outside their region, and only a minority had in-house immunofluorescence (IF) or electron microscopy (EM) services. With the global expansion of telemedicine, these logistical hurdles can be partly mitigated^[25]^. A feasible solution includes establishing local biopsy processing units equipped with digital imaging systems and high-speed internet, enabling remote nephropathologists to assist in diagnosis. This model has been piloted successfully in other LMIC contexts, facilitating prompt, accurate diagnosis without requiring complete local pathology infrastructure.

Nevertheless, access to IF and EM remains severely limited. Approximately one-sixth of participants, and up to one-third in Africa, lacked access to light microscopy or IF – an alarming statistic given that IF is essential for diagnosing immune-mediated glomerulopathies. EM, while not required for all cases, is critical in up to 40% of biopsies, especially in hereditary and complex immune-complex glomerulonephritis (e.g., fibrillary, cryoglobulinemic, and immunotactoid types)^[10–12]^.

### Evolving paradigms in glomerular disease diagnosis

The diagnostic approach to glomerular diseases has evolved significantly from histologic pattern recognition to pathogenesis-informed classification. This transition emphasizes serologic and molecular tests, which can sometimes obviate the need for biopsy altogether. For instance, the KDIGO 2021 guidelines recommend that patients with nephrotic syndrome and positive anti-PLA2R antibodies may be diagnosed with membranous nephropathy without biopsy^[6]^. However, our survey revealed significant deficits in access to these advanced diagnostics. Tests like serum-free light chain assay, C3 nephritic factor, anti-complement factor-H/B, serum immunofixation electrophoresis, and genetic panels are often unavailable. The inability to perform these tests leads to reliance on empirical treatment, frequently involving immunosuppressants, with potentially harmful consequences.

### Treatment gaps and therapeutic disparities

Another concerning finding was the widespread use of empirical immunosuppression due to limited diagnostic clarity. This practice contradicts evidence-based medicine and exposes patients to unnecessary risks. Conversely, high-income countries are increasingly moving toward targeted biologic therapies based on specific biomarkers and molecular targets – deepening the “therapeutic apartheid” between regions^[1,8]^. Moreover, even basic medications such as angiotensin-converting enzyme inhibitors (ACEIs) or angiotensin receptor blockers (ARBs) are unaffordable or unavailable for a significant portion of patients in LMICs. Approximately 20% of respondents reported difficulty maintaining patients on these generic medications.

### Economic burden and out-of-pocket expenditure

Most patients across surveyed regions finance their healthcare through out-of-pocket spending. Surprisingly, this financial burden was consistent across continents, highlighting a widespread failure to incorporate glomerular disease care into national health coverage schemes^[10]^. This neglect persists even as kidney diseases represent a growing contributor to the global burden of non-communicable diseases. With less than 5% of GDP allocated to healthcare in most LMICs, preventive care is rightly prioritized. However, this should include chronic kidney diseases – particularly glomerular diseases – whose late presentation often leads to irreversible kidney failure and higher downstream costs due to dialysis or transplantation^[12]^.

## The way forward

Addressing the diagnostic gaps in glomerular diseases demands a multipronged strategy:

- Workforce Development: Training nephrologists and pathologists locally while ensuring they have the resources and incentives to stay and practice.
- Infrastructure Investment: Support for local processing of biopsy samples, including IF and EM capabilities.
- Telemedicine Integration: Linking centers with limited capacity to regional or international centers of excellence.
- Public–Private Partnerships: Shared diagnostic and therapeutic services between private nephropathology labs and public sector hospitals.
- Policy Inclusion: Advocating for including glomerular disease care in national health insurance programs.
- Affordable Therapeutics: Government bulk procurement of essential medications (e.g., ACEIs, ARBs, immunosuppressants, biologics) to ensure wider access.
- Research Inclusion: Involving LMIC centers in international cohort studies and RCTs to facilitate data generation and capacity building.
- Some practical, low-cost solutions—such as reusing sterilized biopsy needles—have already been implemented in countries like India, Bangladesh, and Nigeria, demonstrating that innovation need not be expensive.

## Concluding remarks

Diagnosing glomerular diseases necessitates a comprehensive, integrated approach that leverages the strengths of clinical evaluation, laboratory testing, and histopathological assessment. Our review underscores the importance of clinical vigilance in identifying syndromic presentations such as nephrotic or nephritic syndrome, supported by targeted laboratory investigations, including urinalysis, serologic markers, and renal function panels. Crucially, renal biopsy with light microscopy, immunofluorescence, and electron microscopy remain the cornerstone for definitive diagnosis, particularly in complex or ambiguous cases.

Synthesis of these three domains – clinical, laboratory, and histopathological – enhances diagnostic accuracy and informs therapeutic decision-making, risk stratification, and prognosis. This triad is indispensable for implementing evidence-based, personalized care in patients with glomerular diseases. In resource-limited settings, however, challenges persist across all three fronts, emphasizing the need for systemic investment in nephrology infrastructure, telemedicine, and capacity building.

Ultimately, our paper validates that no single modality suffices in isolation. Only through integrative diagnostic frameworks can clinicians navigate the complex spectrum of glomerular pathology and optimize outcomes for affected individuals.

## Limitations of the study

Our study has several limitations that should be acknowledged. First, it relied on secondary data sources, which may introduce bias and limit the strength of the conclusions drawn.

Second, the exclusion of non-English studies potentially led to an underrepresentation of perspectives and data from non-English-speaking regions, particularly from low- and middle-income countries.

Third, the study’s findings were partially based on self-reported survey data, which are subject to recall bias and inaccuracies.

Fourth, the absence of longitudinal data or outcome tracking restricts the ability to evaluate long-term diagnostic disparities and their impact on patient outcomes.

Fifth, although practical recommendations were provided, the study lacks detailed implementation strategies and timelines, limiting the direct translation into clinical or policy action.

Sixth, the search strategy did not fully utilize the most authoritative databases, such as PubMed (MEDLINE) and Clarivate (Publons), which may have affected the comprehensiveness and reliability of the literature review.

Seventh, multiple forms of bias – including collection bias, regional bias, language bias, selection bias, database searching bias, and publication bias – may influence the generalizability and validity of the study’s conclusions.

While these limitations do not invalidate the key findings, they underscore the need for cautious interpretation and further primary research to strengthen the evidence base.

## Data Availability

The views and opinions expressed in this paper are solely those of the author and do not necessarily reflect the official policies or positions of any affiliated institution or organization. The author declares no conflicts of interest or financial relationships relevant to this research. All data generated or analyzed during this study are included in this published article and its supplementary information files.
